# Effect of oxaliplatin‐based chemotherapy on chemosensitivity in patients with peritoneal metastasis from colorectal cancer treated with cytoreductive surgery and hyperthermic intraperitoneal chemotherapy: proof‐of‐concept study

**DOI:** 10.1093/bjsopen/zraa075

**Published:** 2021-04-11

**Authors:** A Prabhu, A Brandl, S Wakama, S Sako, H Ishibashi, A Mizumoto, N Takao, M Ichinose, S Motoi, Y Liu, Y Yonemura

**Affiliations:** 1 Department of Surgical Oncology Thangam Cancer Centre, Namakkal India; 2 Digestive Unit Champalimaud Foundation, Lisbon, Portugal; 3 Department of Surgery Graduate School of Medicine, Kyoto University, Kyoto, Japan; 4 Non‐Profit Organization to Support Peritoneal Surface Malignancy Treatment Japanese/Asian School of Peritoneal Surface Oncology, Kyoto Japan; 5 Department of Regional Cancer Therapy Peritoneal Surface Malignancy Centre, Kishiwada, Tokushukai Hospital Kishiwada Japan; 6 Department of Regional Cancer Therapy Peritoneal Surface Malignancy Centre, Kusatsu General Hospital Shiga Japan

## Abstract

**Background:**

Chemosensitivity testing, including collagen gel droplet‐embedded culture drug sensitivity test, has proven to be a useful tool in therapeutic decision‐making. This retrospective analysis investigated chemosensitivity testing of peritoneal metastases collected during cytoreductive surgery (CRS), and its impact on survival in patients with colorectal cancer.

**Methods:**

All patients with peritoneal metastasis from colorectal cancer who underwent CRS with or without hyperthermic intraperitoneal chemotherapy (HIPEC) between November 2008 and October 2014 were included. The growth inhibition rate was expressed as the ratio between the image density after treatment (T) and that before treatment (control, C). Tumours with a reduction in T/C ratio of less than 20 per cent were defined as resistant and those with a reduction of 20 per cent or more as sensitive. Groups were compared for overall (OS) and disease‐free (DFS) survival.

**Results:**

Of 84 eligible patients, 81 received neoadjuvant chemotherapy (NACT), including 56 patients with an oxaliplatin‐based regimen. Mean(s.d.) follow‐up was 23·4(22·9) months. The median overall survival of all patients was 19·0 (i.q.r. 5·7–36·1) months, with a progression‐free survival time of 10·1 (4·5–17·0) months. Patients who received oxaliplatin‐based NACT had significantly altered chemosensitivity to oxaliplatin; only 20 of 51 such patients showed chemosensitivity to oxaliplatin compared with 16 of 24 who did not undergo oxaliplatin‐based NACT (*P* = 0·046). However, patients who showed chemoresistance to oxaliplatin had similar OS to those with chemosensitivity (18·8 *versus* 18·1 months; *P* = 0·835). The choice of HIPEC agents in patients who received oxaliplatin‐based NACT did not significantly influence survival (oxaliplatin *versus* mitomycin C: median OS 20·6 (10·9–24·8) *versus* 19·0 (10·5–34·6) months, *P =* 0·811; DFS 6·6 (2·8–25·7) *versus* 9·3 (4·1–13·9) months, *P =* 0·191).

**Conclusion:**

Patients who had oxaliplatin‐based NACT showed a higher rate of chemoresistance to oxaliplatin at the time of CRS and HIPEC. The impact of chemosensitivity testing on OS remains unclear and needs further investigation.

## Teaser

Patients receiving oxaliplatin‐based neoadjuvant chemotherapy owing to peritoneal metastasis from colorectal cancer showed a higher rate of chemoresistance to oxaliplatin at the time of cytoreductive surgery and hyperthermic intraperitoneal chemotherapy (HIPEC) in this proof‐of‐concept study. Although a survival benefit is yet to be demonstrated, the drug used during HIPEC must be selected carefully, considering the development of chemoresistance in tumour tissue as a result of neoadjuvant treatment.

## Resumen

**Antecedentes:**

El análisis de la quimiosensibilidad, tales como la sensibilidad de los fármacos en cultivos con inclusión de gotas de colágeno ha demostrado ser útil como herramienta para la toma de decisiones. Este análisis retrospectivo investigó la quimiosensibilidad de las metástasis peritoneales extraídas durante la cirugía citorreductora (*cytoreductive surgery*, CRS) y su impacto en la supervivencia de pacientes con cáncer colorrectal.

**Métodos:**

Se incluyeron todos los pacientes con metástasis peritoneales de cáncer colorrectal tratados con CRS y quimioterapia intraperitoneal hipertérmica (*hyperthermic intraperitoneal chemotherapy*, HIPEC) entre noviembre de 2008 y octubre de 2014. La tasa de inhibición del crecimiento se expresó como la relación entre la densidad de la imagen después del tratamiento (T) y la densidad de la imagen antes del tratamiento ‐ grupo control (C)‐: tasa T/C. Los tumores con una reducción de la tasa T/C de < 20% se definieron como quimiorresistentes, mientras que los tumores con T/C ≥ 20% se definieron como quimiosensibles. Se compararon la supervivencia global y la supervivencia libre de enfermedad entre los grupos mediante la prueba del rango logarítmico.

**Resultados:**

De un total de 84 pacientes elegibles, 81 fueron tratados con quimioterapia neoadyuvante, incluyendo 56 pacientes tratados con quimioterapia basada en oxaliplatino. El tiempo medio de seguimiento fue de 23,4 ± 22,9 meses. La mediana de supervivencia global de todos los pacientes fue de 19,0 ± 3,0 meses con una supervivencia libre de enfermedad de 10,1 ± 1,8 meses. Los pacientes tratados con oxaliplatino mostraban una quimiosensibilidad al oxaliplatino alterada (T/C: 20/51 pacientes; 39,2%) en comparación con los pacientes tratados sin oxaliplatino (T/C: 16/24; 67%; *P* = 0,02). Sin embargo, los pacientes co quimiorresistencia al oxaliplatino presentaron una supervivencia global similar a los pacientes quimiosensibles (20,6 *versus* 18,1 meses; *P* = 0,89). La elección de los fármacos para la HIPEC en los pacientes tratados con quimioterapia neoadyuvante basada en oxaliplatino no mostró influencia en la supervivencia global ni en la supervivencia libre de enfermedad cuando se comparaba con HIPEC con oxaliplatino o mitomicina C (20,6 ± 3,1 *versus* 15,3 ± 2,4 meses; *P* = 0,62; mediana supervivencia libre de enfermedad 6,6 ± 8,1 *versus* 9,3 ± 1,9 meses; *P* = 0,18).

**Conclusión:**

Los pacientes tratados con quimioterapia neoadyuvante basada en oxaliplatino mostraron una tasa más alta de quimiorresistencia al oxaliplatino en la CRS e HIPEC. No se ha observado impacto en la supervivencia, pero este aspecto debe ser investigado especialmente.

Chemoresistance and HIPEC in colorectal metastasis

## Introduction

Patients with colorectal cancer and peritoneal metastasis (PM) as the only site of metastasis represent approximately 25–35 per cent of all patients with stage IV colorectal cancer^1^. Cytoreductive surgery (CRS) and hyperthermic intraperitoneal chemotherapy (HIPEC) improve survival of these patients^2–6^. A number of studies^7–9^ have reported median overall survival (OS) ranging from 22 to 63 months in selected patients, compared with only 10–28 months after systemic chemotherapy alone.

However, several aspects in the selection of treatment regimens, dose, dialysate, temperatures and duration of HIPEC have yet to be standardized. Treatment protocols differ between groups and a large number of patients needs to be included in clinical studies for a statistically significant difference to be shown; thus, there are various confounding factors making it difficult to draw firm conclusions.

Chemosensitivity testing has been used to guide treatment for several cancers, including colorectal cancer. The collagen gel droplet‐embedded culture drug sensitivity test (CD‐DST) is an *in vitro* anticancer drug sensitivity test in which even small quantities of tumour cells can be cultured successfully, while maintaining the original growth characteristics of the cancer cells. This technique has the ability to evaluate chemosensitivity to various chemotherapeutic drugs at physiological concentrations^10^.

A previous study^11^ reported on the clinical efficacy and feasibility of CD‐DST in guiding systemic treatment in patients with stage IV colorectal cancer. Reported radiological response rates were 85·7 per cent in patients treated with drugs that showed sensitivity on *in vitro* chemosensitivity testing, compared with 41·6 per cent in those treated with drugs that were non‐sensitive *in vitro* (*P* < 0·001). Patients benefiting from optimization of the chemotherapy regimen by means of *in vitro* drug sensitivity assessments experienced an improved median progression‐free survival (PFS) of 23 months and a median OS of 34 months, compared with 10 months (*P =* 0·03) and 17 months (*P =* 0·001) respectively in patients who did not undergo chemosensitivity testing. Other reports^12–15^ have described the value of CD‐DST‐guided choice of chemotherapeutic agents in gastric, ovarian, lung and breast cancers, leading to a survival benefit for patients.

The aim of this retrospective analysis was to evaluate the chemosensitivity of PMs collected during CRS in patients with colorectal cancer, and to assess its association with survival.

## Methods

Chemosensitivity testing (CD‐DST) of tumour tissue collected during CRS and before HIPEC has been done routinely since November 2008 in the peritoneal surface malignancy unit of Tokushukai Hospital, Kishiwada, and Kusatsu General Hospital, Shiga, Japan. The unit is a national referral centre for peritoneal malignancies, performing more than 200 cytoreductive procedures per year, and is part of the Japanese/Asian School of Peritoneal Surface Oncology.

All patients with histologically proven PM from colorectal cancer treated with CRS with or without HIPEC, between November 2008 and October 2014 were reviewed. Those who had undergone CD‐DST of tumour tissue were included in this study. CD‐DST was undertaken for patients whose treatment‐related expenses were covered by their health insurance or if it was paid out of pocket. Patients who underwent standard perioperative chemotherapy, and CRS with or without HIPEC, who did not have CD‐DST, were excluded from the present analysis.

The database contained demographic, operative (Peritoneal Carcinomatosis Index, blood loss, duration of surgery, completeness of cytoreduction (CC) score) and histopathological data. Neoadjuvant, intraoperative and adjuvant chemotherapy regimens were noted, including dose, number of cycles and therapeutic agents used. Postoperative complications were recorded up to a minimum of 30 days or until the day of discharge. Complications were graded according to the Clavien–Dindo classification^16^. Major postoperative morbidity was defined as grade III and grade IV complications. Follow‐up was carried out routinely at the outpatient clinic in the authors' institute, including clinical examination, analysis of tumour markers (carcinoembryonic antigen, carbohydrate antigen (CA) 125 and CA‐19·9) and CT annually, or earlier if indicated clinically. Clinical follow‐up was undertaken every 2 months in the first 2 years and 6‐monthly thereafter.

### Chemotherapy regimens

Neoadjuvant chemotherapy (NACT) included FOLFOX (5‐fluorouracil (5‐FU), leucovorin, oxaliplatin), FOLFIRI (5‐fluorouracil, leucovorin, irinotecan), XELOX (capecitabine, oxaliplatin), XELIRI (capecitabine, irinotecan), IRIS (irinotecan, S1) and SOX (S1, oxaliplatin). On average, patients were treated with five or six cycles of NACT before restaging and further evaluation. Response evaluation was accomplished by CT, and patients with a clinical, radiographic or biochemical (tumour markers) response or stable disease were selected for CRS and HIPEC. Adjuvant chemotherapy was started 4–6 weeks after CRS and HIPEC; the drugs were chosen based on the results of *in vitro* CD‐DST of tumour tissue collected during surgery.

The following drugs and concentrations were used during HIPEC: cisplatin 40 mg/m^2^, oxaliplatin 200 mg/m^2^, mitomycin C 20 mg/m^2^ and 5‐FU 500 mg/m^2^. HIPEC was administered by an open technique and for a duration of 40 min at a temperature of 43°C. Drugs were chosen at the surgeon's discretion or according to the treatment policy being followed at the time. HIPEC was avoided in patients with a difficult intraoperative course, such as excessive blood loss, or inability to maintain adequate urine output or mean arterial pressures. Bowel anastomosis was performed after completion of HIPEC.

**Fig. 1:**
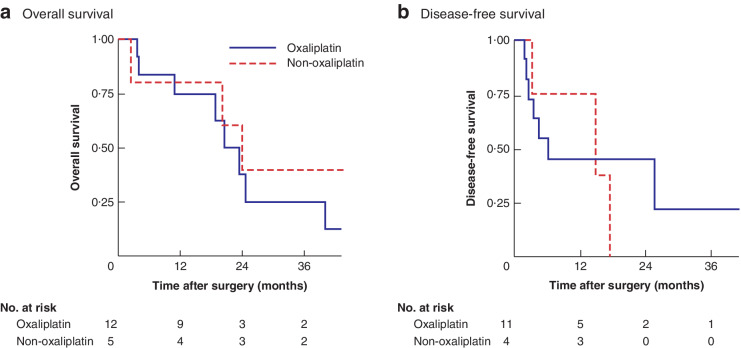
Overall and disease‐free survival in patients treated with oxaliplatin hyperthermic intraperitoneal chemotherapy according to type of neoadjuvant chemotherapy

**a** Overall and **b** disease‐free survival. **a***P* = 0·721, **b***P* = 0·856 (log rank test).

### Collagen gel droplet‐embedded culture drug sensitivity test

Tumour tissue obtained during CRS and before HIPEC was minced finely with a scalpel and digested using collagenase (Kurabo Industries, Osaka, Japan)^17^. The tumour cells were incubated in a collagen gel‐coated flask (CG flask; Kurabo Industries) at 37°C for 24 h. Viable cells adhering to the collagen gel were used for chemosensitivity testing. A cancer cell suspension was created by adding these viable cells to reconstructed type I collagen solution (Cellmatrix® Type CD; Kurabo Industries) and three drops of this cell suspension were then added to each well of a six‐well plate. Oxaliplatin (0·5 μg/ml), mitomycin C (MMC) (2 μg/ml), 5‐FU (1 μg/ml) and irinotecan (0·03 μg/ml) were added to separate wells and incubated for 24 h. Not all patients underwent CD‐DST for all four drugs, as the number of tumour cells harvested before culture differed significantly between patients. If there were not enough cells, fewer drugs were tested, with the choice depending on the numbers of cells and type of NACT. After 24 h, the medium containing anticancer drug was removed, and each well was rinsed with 3 ml Hank's balanced salt solution and further incubated in PCM‐2 medium (Kurabo Industries) for 7 days. Neutral red was added to each well after 7 days of incubation to obtain a final concentration of 50 μg/ml. The colonies in the collagen gel droplet were stained for 2 h, fixed in 10 per cent buffered formalin, washed in water, air‐dried and quantified by image analysis^18–20^. Image density was also analysed on day 0, before the chemotherapeutic agent had been added.

### Analysis of growth inhibition rate

The growth inhibition rate was calculated using image density (total volume of cells observed) on day 7 *versus* day 0. The growth inhibition rate or the *in vitro* chemosensitivity effect was expressed as the T/C ratio, where T represents the image density of the treated group and C the image density of the cells on day 0 (considered as the control group). Tumours with a reduction in T/C ratio of below 20 per cent were considered resistant and those with a value of 20 per cent or more as sensitive. Although T/C ratios of 60 or 50 per cent were taken to indicate sensitivity in a previous study^11^ of metastatic colorectal cancer, these tests were carried out on chemotherapy‐naive cells, whereas patients in the present study received preoperative chemotherapy.

### Statistical analysis

Continuous and categorical data were compared using the *t* test and χ^2^ test respectively. OS was calculated from the date of surgery (CRS and HIPEC) to the date of death or last follow‐up. Disease‐free survival (DFS) was defined as the time until tumour recurrence, and calculated for patients undergoing CC0/1 procedures, from the date of surgery (CRS and HIPEC) to the date of recurrence or death. PFS was defined as tumour progression (in patients undergoing CC2 or CC3 procedures) or tumour recurrence (in patients having CC0–1 surgery) and calculated from the date of surgery (CRS and HIPEC) to the date of tumour progression (CC2 or CC3), tumour recurrence (CC0–1) or death. Survival analysis was done using the Kaplan–Meier method, with differences between groups evaluated by means of the log rank test. *P* < 0·050 was considered statistically significant. All statistical analyses were performed using the SPSS® software version 22 (IBM, Armonk, New York, USA).

## Results

Of 190 patients who underwent CRS with or without HIPEC during the study interval, 84 (44·2 per cent) underwent CD‐DST. Overall, 81 received NACT, 56 patients being treated with oxaliplatin‐based NACT. Three patients did not have NACT, but underwent chemosensitivity testing, as it influenced the choice of adjuvant chemotherapy. These oxaliplatin‐naive patients were included in the group without oxaliplatin‐based NACT in further analyses.

Baseline demographic, clinicopathological, and treatment characteristics are shown in *Table* *1*. The most common histopathological subtype was adenocarcinoma (54 of 83, 65 per cent) and sigmoid colon was the most common site of the primary tumour (24 of 83, 29 per cent). Complete macroscopic cytoreduction (CC0–1) was achieved in 64 patients (76 per cent). The postoperative major morbidity rate was 22 per cent and the postoperative mortality rate 2 per cent.

**Fig. 2:**
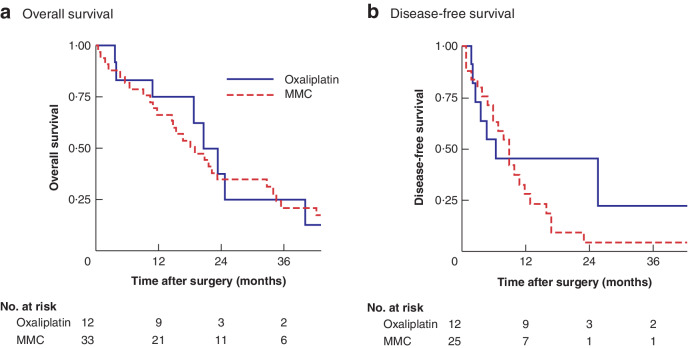
Overall and disease‐free survival in patients who had oxaliplatin neoadjuvant chemotherapy according to type of hyperthermic intraperitoneal chemotherapy

**a** Overall and **b** disease‐free survival. MMC, mitomycin C. **a***P* = 0·623, **b***P* = 0·183 (log rank test).

**Fig. 3:**
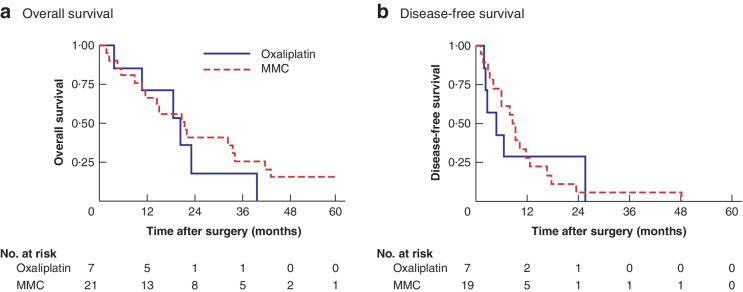
Overall and disease‐free survival in patients without sensitivity to oxaliplatin according to type of hyperthermic intraperitoneal chemotherapy

**a** Overall and **b** disease‐free survival. MMC, mitomycin C. **a***P* = 0·897, **b***P* = 0·808 (log rank test).

**Table 1:** Baseline demographic, clinicopathological and treatment characteristics of 84 patients with colorectal cancer and peritoneal metastasis treated with cytoreductive surgery, with or without hyperthermic intraperitoneal chemotherapy

	No. of patients (*n* = 84)	Missing values
**Age (years)** *	52·5(13·1)	0
**Sex ratio (M** : **F)**	45 : 39	
**Primary tumour location**		1
Caecum	19 (23)	
Ascending colon	7 (8)	
Transverse colon	10 (12)	
Descending colon	6 (7)	
Sigmoid colon	24 (29)	
Rectosigmoid	17 (20)	
**Histopathological type**		1
Adenocarcinoma	54 (65)	
Mucinous	11 (13)	
Signet ring cell	18 (22)	
**NACT**		0
Oxaliplatin‐based	56 (67)	
Non‐oxaliplatin‐based	25 (30)	
No NACT	3 (4)	
**PCI score** *		0
Total PCI	13·2(9·6)	
Small bowel PCI	3·5(3·3)	
**HIPEC**		0
Yes	64 (76)	
No	20 (24)	
**HIPEC drug**		0
Cisplatin + MMC	44 (69)	
Oxaliplatin + 5‐FU	17 (27)	
MMC	2 (3)	
Cisplatin + 5‐FU	1 (2)	
**Duration of surgery (min)** *	283(89)	0
**Mean blood loss (ml)** *	1819(975)	0
**Amount of blood transfused (units)** *	6·0(5·1)	0
**Completeness of cytoreduction score**	0
CC0	58 (69)	
CC1	6 (7)	
CC2	9 (11)	
CC3	11 (13)	
**Postoperative complications (Clavien–Dindo grade)**	2
0	45 (55)	
I–II	17 (21)	
IIIa	9 (11)	
IIIb	3 (4)	
IV	6 (7)	
V	2 (2)	

Values in parentheses are percentages unless indicated otherwise;

* values are mean(s.d.). NACT, neoadjuvant chemotherapy; PCI, Peritoneal Carcinomatosis Index; HIPEC, hyperthermic intraperitoneal chemotherapy; MMC, mitomycin C; 5‐FU, 5‐fluorouracil.

### Chemosensitivity

The mean(s.d.) reduction in tumour density was 19·3(11·5) per cent for oxaliplatin and 24·7(15·6) per cent for MMC. Analysis of the chemosensitivity of tumour tissue to oxaliplatin revealed that patients who received oxaliplatin‐based NACT had significantly altered chemosensitivity; tissue from only 20 of 51 patients who received oxaliplatin‐based NACT was sensitive to oxaliplatin, compared with 16 of 24 who did not have oxaliplatin‐based NACT (*P =* 0·046). In contrast, patients treated with or without oxaliplatin‐based NACT had similar results in terms of chemosensitivity to MMC (20 of 40 *versus* 11 of 18; *P =* 0·571). Analysis of chemosensitivity to both agents (MMC and oxaliplatin) showed that tumour tissue that was not chemosensitive to oxaliplatin was also likely not to be chemosensitive to MMC, although the effect was not significant (*P =* 0·414) (*Table* *2*). A comparison of demographic, operative and therapeutic factors in groups with chemosensitivity and chemoresistance to oxaliplatin is shown in *Table* *3*. These groups differed significantly only in terms of the rate of complete macroscopic cytoreduction (22 of 36 patients with oxaliplatin‐sensitive tumours compared with 33 of 39 in the oxaliplatin‐resistant group; *P =* 0·035). Comparison of similar factors between patients who did or did not receive oxaliplatin‐based NACT showed no significant differences (*Table* *4*).

**Table 2:** Chemosensitivity to oxaliplatin or mitomycin C in relation to receipt of oxaliplatin‐based neoadjuvant chemotherapy

	Chemosensitivity	
	No	Yes	*P* §
**Chemosensitivity to oxaliplatin** *			0·046
Oxaliplatin‐based NACT	31 of 51 (61)	20 of 51 (39)	
Non‐oxaliplatin‐based NACT	8 of 24 (33)	16 of 24 (67)	
**Chemosensitivity to MMC** **†**			0·571
Oxaliplatin‐based NACT	20 of 40 (50)	20 of 40 (50)	
Non‐oxaliplatin‐based NACT	7 of 18 (39)	11 of 18 (61)	
**Chemosensitivity to MMC** **‡**			0·414
Chemosensitive to oxaliplatin	11 of 27 (41)	16 of 27 (59)	
Not chemosensitive to oxaliplatin	15 of 27 (56)	12 of 27 (44)	

Values in parentheses are percentages.

* Five patients in the oxaliplatin‐ and four in the non‐oxaliplatin‐based neoadjuvant chemotherapy (NACT) group did not undergo chemosensitivity testing for oxaliplatin.

† Sixteen patients in the oxaliplatin‐ and ten patients in the non‐oxaliplatin‐based NACT group did not undergo chemosensitivity testing for mitomycin C (MMC).

‡ Nine patients with and 12 without chemosensitivity to oxaliplatin did not undergo chemosensitivity testing for MMC; three patients with and one without chemosensitivity to MMC did not undergo chemosensitivity testing for oxaliplatin.

§ χ^2^ test.

**Table 3:** Demographic, clinicopathological and treatment characteristics in relation to sensitivity to oxaliplatin

	Without chemosensitivity to oxaliplatin (*n* = 39)	Chemosensitivity to oxaliplatin (*n* = 36)	*P* †
**Age (years)** *	53·5(13·6)	50·2(12·6)	0·270‡
**Sex ratio (M** : **F)**	23 : 16	18 : 18	0·491
**Primary tumour location**			0·815
Right	16	16	
Left	23	19	
Missing	0	1	
**CEA (ng/ml)** *	54·5(124·3)	88·4(227·5)	0·427‡
**PCI score** *			
Total PCI	12·5(9·4)	14·9(10·0)	0·288‡
Small bowel PCI	3·3(3·0)	3·7(3·8)	0·576‡
**Completeness of cytoreduction score**			0·035
CC0–1	33	22	
CC2–3	6	14	
**Histopathological type**			0·787
Adenocarcinoma	24	23	
Mucinous	6	5	
Signet ring cell	9	7	
Missing	0	1	
**Blood loss (ml)** *	1687(961)	1877(1062)	0·441‡
**Duration of surgery (min)** *	304(88)	269(92)	0·116‡
**No. of cycles of NACT** *	6·4(2·2)	8·2(5·2)	0·356‡
**Adjuvant chemotherapy**	27 of 27	20 of 20	

* Values are mean(s.d.). CEA, carcinoembryonic antigen; PCI, Peritoneal Carcinomatosis Index; NACT, neoadjuvant chemotherapy.

† χ^2^ test, except

‡ *t* test.

**Table 4:** Demographic, clinicopathological and treatment characteristics in patients who received neoadjuvant chemotherapy with or without oxaliplatin

	Oxaliplatin‐based NACT (*n* = 56)	Non‐oxaliplatin‐based NACT (*n* = 28)	*P* †
**Age (years)** *	51·8(13·6)	53·9(12·2)	0·491‡
**Sex ratio (M** : **F)**	29 : 27	16 : 12	0·817
**Primary tumour location**			0·638
Right	23	13	
Left	33	14	
Missing	0	1	
**CEA (ng/ml)** *	58·3(169·9)	85·1(179·9)	0·512‡
**PCI score** *			
Total PCI	13·5(10·3)	12·7(8·2)	0·732‡
Small bowel PCI	3·6(3·3)	3·4(3·3)	0·729‡
**Completeness of cytoreduction score**			0·102
CC0–1	46	18	
CC2–3	10	10	
**Histopathological type**			0·816
Adenocarcinoma	36	18	
Mucinous	8	3	
Signet ring cell	12	6	
Missing	0	1	
**Blood loss (ml)** *	1756(939)	1950(1053)	0·420‡
**Duration of surgery (min)** *	287(88)	277(94)	0·669‡
**No. of cycles of NACT** *	7·1(3·5)	4·5(2·1)	0·342‡
**Adjuvant chemotherapy**	37 of 41	20 of 22	1·000

* Values are mean(s.d.). CEA, carcinoembryonic antigen; PCI, Peritoneal Carcinomatosis Index; NACT, neoadjuvant chemotherapy.

† χ^2^ test, except

‡ *t* test.

### Survival

Mean(s.d.) follow‐up was 23·4(22·9) months. Median OS for all patients was 19·0 (i.q.r. 5·7–36·1) months, with a PFS of 10·1 (4·5–17·0) months. Analysis according to the type of NACT showed no difference between patients treated with *versus* without oxaliplatin‐based NACT, with both groups receiving oxaliplatin HIPEC; median overall survival was 20·6 (10·9–24·8) *versus* 24·0 (20·0 to not reached) months respectively (*P =* 0·721), and DFS was 6·6 (2·8–25·7) *versus* 15·0 (3·5–17·8) months (*P =* 0·856) (*Fig*. *1*). Among patients treated with oxaliplatin NACT, there was no significant difference in OS between those who presented with chemoresistance to oxaliplatin and patients who retained chemosensitivity to oxaliplatin (18·8 (5·7–34·0) *versus* 18·1 (6·5–35·5) months respectively; *P =* 0·835). Thus, OS was not influenced by whether the NACT included oxaliplatin, or by chemosensitivity to oxaliplatin.

Factors influencing DFS were evaluated in univariable analysis (*Table* *5*). Positive intraoperative cytology (*P =* 0·002) and chemosensitivity to MMC (*P =* 0·032) were the only two significant predictors.

**Table 5:** Univariable analyses of factors affecting disease‐free survival of patients with colorectal cancer and peritoneal metastasis after cytoreductive surgery with or without hyperthermic intraperitoneal chemotherapy

	No. of patients	Disease‐free survival (months)*	*P*†
**Age (years)**			0·620
≤ 60	46	9·5 (6·5–12·5)	
> 60	15	12·7 (1·9–23·5)	
**Sex**			0·061
M	32	12·7 (7·8–17·6)	
F	29	8·6 (5·3–11·9)	
**Primary tumour location**			0·177
Right	24	10·4 (4·6–16·2)	
Left	36	6·6 (1·6–11·6)	
**Lymph node status**			0·156
Negative	28	8·6 (6·5–10·7)	
Positive	31	10·3 (6·0–14·6)	
**Histopathological type**			0·085
Adenocarcinoma	49	10·3 (5·8–14·8)	
Signet ring cell	12	4·5 (2·6–6·4)	
**Total PCI score**			0·168
0–15	45	11·8 (7·1–16·5)	
> 15	16	7·9 (4·5–11·3)	
**Small bowel PCI**			0·082
≤ 2	32	12·7 (8·1–17·3)	
> 2	29	7·9 (4·2–11·6)	
**Liver metastasis**			0·048
No	44	11·8 (8·1–16·5)	
Yes	17	6·0 (2·3–9·7)	
**HIPEC**			0·078
Oxaliplatin	15	15·0 (0–31·2)	
MMC	32	9·3 (7·2–11·4)	
**Intraoperative cytology**			0·002
Negative	34	13·0 (9·1–16·9)	
Positive	17	4·1 (2·5–5·7)	
**NACT**			0·397
Oxaliplatin‐based	17	14·8 (11·7–17·9)	
Non‐oxaliplatin‐based	44	8·6 (5·1–12·1)	
**Chemosensitive to oxaliplatin**			0·619
No	32	9·3 (6·3–12·3)	
Yes	21	9·5 (0·4–18·6)	
**Chemosensitivity to MMC**			0·032
No	20	8·6 (0·9–16·3)	
Yes	21	15·0 (4·9–25·1)	

*Values are median (95% confidence intervals). PCI, Peritoneal Carcinomatosis Index; HIPEC, hyperthermic intraperitoneal chemotherapy; NACT, neoadjuvant chemotherapy; MMC, mitomycin C. †Log rank test (univariable analysis).

In an analysis including all patients treated with oxaliplatin NACT, no significant difference in OS or DFS was noted after HIPEC with oxaliplatin *versus* MMC (median OS 20·6 (10·9–24·8) *versus* 19·0 (10·5–34·6) months, *P =* 0·811; DFS 6·6 (2·8–25·7) *versus* 9·3 (4·1–13·9) months, *P =* 0·191) (*Fig*. *2*). Among patients whose tumours showed chemoresistance to oxaliplatin after oxaliplatin NACT, OS and DFS were no different following intraoperative HIPEC with oxaliplatin *versus* MMC (median OS 20·6 (10·9–23·4) *versus* 21·6 (10·8–42·2) months, *P =* 0·444; DFS 4·8 (2·2–25·7) *versus* 9·3 (4·1–12·7) months, *P =* 0·924) (*Fig*. *3*).

## Discussion

This single‐institution observational study of prospectively collected data from a high‐volume centre focused on the impact of chemosensitivity testing on the choice of postoperative chemotherapy and the effect of intraoperative HIPEC. The results are important, especially in the context of the negative findings of the PRODIGE 7 RCT^21^, in which patients who had CRS for PM from colorectal cancer were randomized to HIPEC or no HIPEC. This French multicentre trial showed no significant difference between patients treated with CRS and HIPEC and those who had CRS alone. Despite the main shortcoming of an underpowered analysis owing to the underestimated OS in the control group (without HIPEC), one major doubt concerns the duration and dose of HIPEC. As preclinical data are limited to a few publications in experimental models^22^, clinical observations about chemosensitivity and therefore potential optimization of the chemotherapeutic drug seem promising.

The present study was able to show, as a proof of concept, that chemoresistance to oxaliplatin might be influenced by the preoperative chemotherapy regimen. Patients who had oxaliplatin‐based NACT had significantly higher rates of chemoresistance to oxaliplatin than patients who did not receive oxaliplatin‐based NACT. Previous investigations have reported the development of chemoresistance in cell lines after treatment with oxaliplatin, through molecular mechanisms such as upregulation of microRNA (mir 203), and downregulation of ataxia telangiectasia mutated mRNA and protein levels^23^. A recent study^24^ demonstrated that the induction of circCCDC66 is dependent on treatment with oxaliplatin and is required for the establishment of chemoresistance to this agent. However, another study^25^ reported on the *ex vivo* activity of chemotherapeutic drugs in patients with PM from various cancers, including colorectal cancer, using a different method for drug testing sensitivity, the fluorometric microculture cytotoxic assay, and the authors demonstrated that oxaliplatin was equally active in chemotherapy‐naive and previously treated patients. Data focusing on the optimization of chemotherapy regimens guided by chemosensitivity testing are seen as controversial, as increased response rates do not necessarily translate into increased survival. A recent study^26^ revealed no survival benefit for patients with unresectable colorectal cancer and CD‐DST‐guided first‐line chemotherapy compared with patients who received standard first‐line chemotherapy in a cohort of 120 patients. Interestingly, the subgroup of poor responders to chemotherapy showed significantly improved survival if treated with CD‐DST‐guided first‐line chemotherapy. Despite this limited evidence, there is a considerable lack of clinical trials, which are necessary to support a tailored chemotherapy strategy.

Although patients appeared to develop chemoresistance during oxaliplatin‐based NACT, the clinical outcome, especially OS, was not affected by this finding. Interestingly, neither the choice of HIPEC (oxaliplatin‐ *versus* non‐oxaliplatin‐based), nor receiving oxaliplatin‐based NACT, had an effect on OS. These results might be explained by several factors, including small sample size. First, the chemoresistant group had a significantly higher rate of complete cytoreductions. As the completeness of cytoreduction is one of the most important factors predicting patient outcome and therefore OS^27^, inferior OS would be expected for the group showing chemosensitivity to oxaliplatin; however, this was not the case, suggesting that survival would hypothetically be better in this group after equal surgical treatment. Second, the authors' clinical practice of choosing the adjuvant chemotherapy regimen according to the results of chemosensitivity testing might have had an influence on outcomes, as patients in whom chemoresistance was detected were treated with adjuvant drugs that attacked the tumour precisely owing to its sensitivity. It is known that chemotherapy with the choice of drugs influenced by chemosensitivity testing is more effective than a rather blind choice of regimen^11^. Third, the effect of the drug distributed during HIPEC may have a minor effect on OS. This hypothesis is supported by the negative results of PRODIGE 7^21^, in which OS was similar for patients with PM from colorectal cancer treated with CRS *versus* CRS and HIPEC. Unfortunately, no subgroup analysis comparing patients treated with or without NACT is yet available. In contrast to this hypothesis, another RCT^28^ proved the superiority of CRS and HIPEC over intravenous chemotherapy, and further large retrospective trials^4,29^ have also shown increased OS among patients selected for CRS and HIPEC. A well conducted clinical study with a sample size calculation may help in understanding the effect of chemoresistance due to NACT on OS.

This study has a few limitations. The cut‐off of 20 per cent reduction in cell numbers after 7 days has not been validated previously, as the majority of studies of CD‐DST used either immortalized cell lines or chemotherapy‐naive tumour tissue. The established cut‐off values of 50–60 per cent are based on these colorectal cell types, and represent a different situation with a different opportunity for chemotherapeutic agents to act. Studies of different tumour entities, for example pancreatic cancer, revealed an optimal tumour reduction of 15 per cent indicating overall recurrence, with sensitivity and specificity of 61·1 and 100 per cent respectively^30^. The range of cell density in the present assay, with a mean reduction in tumour density of 19·3 and 24·7 per cent for oxaliplatin and MMC respectively, was lower than that in previous series^11^. Therefore, changing the cut‐off value from 50 to 20 per cent for the pretreated tumour tissue seemed necessary, although it was not based on existing evidence. Another limitation is the CD‐DST assay used and the concentration of chemotherapeutic drugs. An incubation period of 24 h before removal of the agent and culturing for an additional 6 days was used. This protocol was described previously and validated for 5‐FU, irinotecan and oxaliplatin in a group with stage IV colorectal cancer^11^, but differs from other published protocols^31^. The concentrations of cytotoxic agents reported here were approximately ten times lower for MMC (2 *versus* 12–25 μg/ml) and about 300 times lower for oxaliplatin (0·5 *versus* 160–330 μg/ml), which is justified by the cytotoxicity associated with the longer incubation time (24 h *versus* 30 min). In addition, possible drug combinations in the CD‐DST assay were not assessed, and only single‐agent incubations were tested, as intraperitoneal MMC or oxaliplatin comprise the majority (76 per cent) of reported HIPEC regimens, and combinations of MMC with cisplatin or oxaliplatin with irinotecan are less common^32^. Furthermore, *ex vivo* testing has a sensitivity and specificity of 90 and 70 per cent respectively, which might also be influenced by the sample of PM chosen, as well as tumour regression after preoperative chemotherapy^33^. Finally, this study did not show a significant impact of chemosensitivity testing on OS, but the present findings provide an incentive to perform a clinical trial with a sufficient sample size to substantiate the hypothesis generated and confirm these clinical findings.

## Disclosure

The authors declare no conflict of interest.

##  

*Funding information*

No funding
